# Uncertainty tolerance in healthcare: towards a normative conception

**DOI:** 10.1007/s11017-025-09731-4

**Published:** 2025-10-15

**Authors:** Paul K. J. Han, Bjørn Hofmann

**Affiliations:** 1 Division of Cancer Control and Population Sciences, National Cancer Institute, National Institutes of Health (NIH), 9609 Medical Center Drive, 3E138, Bethesda, MD 20892, USA; 2 Centre of Medical Ethics, University of Oslo, Oslo, Norway; 3 Institute for the Health Sciences at the Norwegian University of Science and Technology (NTNU) at Gjøvik, Gjøvik, Norway

**Keywords:** Uncertainty, Uncertainty tolerance, Virtues, Framework

## Abstract

This paper critically analyzes the meaning of uncertainty tolerance (UT), a phenomenon of growing interest in healthcare. Medical practitioners, educators, and researchers have increasingly acknowledged the importance of UT for both clinicians and patients, and called for greater attention to improving it. However, we argue that the prevailing conception of UT is an inadequate normative ideal, due to its narrow understanding of uncertainty as exclusively an aversive state entailing negative outcomes, and of tolerance as merely the endurance of these outcomes. We show how this endurance-based, outcomes-focused conception of UT is both theoretically incoherent and practically unhelpful. We make the case for an alternative conception based not on endurance but adaptation, and focused not on outcomes but moral virtues, which we view as instrumental capacities that enable adaptation. We develop a provisional integrative taxonomy of these key virtues, discuss both the promises and challenges of this new adaptation-based, virtue-focused conception of UT, and identify fruitful directions for future work.

## Introduction

‘Uncertainty tolerance’ (UT) is a phenomenon that has attracted increasing attention in medicine. Over the past several years, commentaries in major medical journals have called out UT as a “necessary skill for effective care and self-preservation” [1, p. 1666]] and “the next revolution” [2, p. 1713] in healthcare. Building on seminal work of Gerrity [3] and Geller [4, 5], researchers have developed and administered multiple measures of healthcare-specific UT to health professionals, trainees, and patients in a growing number of studies, and generated an expanding evidence base on the relationship between UT, clinician and patient behaviors, and healthcare-related outcomes [6, 7]. This research has provided empirical support for a commonly held view that low UT (uncertainty intolerance) contributes to negative outcomes including overuse of medical interventions and diminished well-being of patients and clinicians [7–9]. Reflecting and reinforcing this view, medical educators have identified UT as a key learning goal and begun to develop teaching interventions aimed at increasing UT [8, 10–12]. Geller has further advocated for the potential use of UT measures in the medical school admissions process, as a means of selecting more uncertainty-tolerant future physicians [13].

Yet despite growing attention and interest in the phenomenon, some scholars have expressed concerns about making UT a more central focus—and normative goal—of medical practice and education. Hancock and Mattick have pointed out the lack of firm empirical evidence on the evolution of UT during medical training, the potential context-dependency of UT and its positive or negative effects for different clinicians, and possible “unintended consequences” of increasing UT for the medical workforce [14]. Ilgen has raised questions about the stability of UT in individuals facing different situations and further argued that as measured by existing instruments, UT may simply be an “epiphenomenon”—a marker of an underlying “competence in context” that emerges as clinicians develop proficiency in dealing with uncertainty and managing complex clinical cases [15]. Haas and Stojan have pointed out how “intolerance” of uncertainty can have desirable effects, such as stimulating curiosity, research, and innovation [16]. In a similar vein, Reis-Dennis and colleagues have argued that UT can be either desirable or undesirable depending on the clinical context; uncertainty tolerance and intolerance represent opposing “fight” vs. “flight” responses that may be either appropriate or inappropriate for clinicians [17]. Referencing the thinking of moral philosopher Philippa Foot, they have further argued that this dual nature of UT raises a need for clinicians and trainees to employ “corrective virtues,” consisting of courage, diligence, and curiosity, that can enable them to avoid being either too tolerant or too intolerant of uncertainty in different clinical circumstances.

Having critically analyzed the construct of UT [6, 7] and put forth our own virtue-focused conception [18, 19], we think these assessments are on the right track in questioning UT as a normative ideal in medical practice and education. However, we believe they do not go far enough, and in the current paper we will attempt to show why. We will critically examine the prevailing concept of UT and the normative assumptions it tacitly reflects and reinforces. We will argue that the problem with UT as traditionally conceptualized extends beyond the lack of empirical evidence on its stability, evolution, or effects on clinicians and patients, or the context-dependency of these effects. We will show that the more fundamental problem with the prevailing conception of UT lies in its inherent consequentialism—i.e., its focus on outcomes and their endurance as the defining feature of UT. We will demonstrate why this endurance-based, outcomes-focused conception of UT is inadequate as a normative ideal, and will make the case for an alternative conception based not on endurance but adaptation, and focused not on outcomes but moral virtues, which we view as instrumental capacities that enable adaptation. We argue that virtues are the essential elements of UT and a useful guiding ideal in situations of uncertainty; they specify who and how to be when what will happen and what to do are unknown and unknowable, and thus provide a needed practical focus for our efforts to manage uncertainty in healthcare. We develop a provisional integrative taxonomy of key uncertainty-tolerant virtues as the foundation of a new adaptation-based, virtue-focused conception of UT, and conclude by briefly discussing key promises and challenges of adopting this conception as a normative ideal in healthcare, and identifying fruitful directions for future work.

## Uncertainty tolerance as a descriptive concept

The first task in our analysis is to examine prevailing conceptions of UT and their normative implications. In this task we turn to the social sciences, where the concept of UT has been empirically investigated primarily in the disciplines of psychology, behavioral economics, and sociology. Researchers in these disciplines have mainly construed UT as a personal characteristic or trait that predisposes individuals towards particular responses to uncertainty, although some researchers have also viewed UT as a psychological state determined by individual and contextual factors [20, 21]. This work has produced several measures of UT and empirical evidence on its effects.

An exhaustive synthesis of this research is beyond the scope of our analysis, and several useful literature reviews exist [22–27]. Our aim is to briefly summarize the main ways UT has been conceptualized based on our previously published conceptual analysis of existing UT measures [6]. This analysis revealed a lack of explicit, precise, consistent, and coherent definitions of either the “uncertainty” or the “tolerance” components of UT. Most existing UT definitions fail to explicitly specify either the *objects* of tolerance (i.e., what uncertainties are being tolerated) or its defining *features or manifestations* (i.e., what human responses to uncertainty constitute tolerance). Existing definitions that do specify these components demonstrate substantial variability. For example, definitions of the objects of tolerance range from “the notion that negative events may occur and there is no definitive way of predicting such events,” [28, p. 106] to information marked by “vague, incomplete, fragmented, multiple, probable, unstructured, uncertain, inconsistent, contrary, contradictory, or unclear meanings” [29, p. 608], to situations that are “unfamiliar or not easily resolved” [30, p. 726]. Definitions of the manifestations of tolerance also span a wide range from less negative to more positive cognitive appraisals, emotions, and attitudes [6, 24, 27].

A similar lack of conceptual specificity characterizes the individual items comprising existing UT measures. Regarding the *objects* of tolerance, measure items often use different terms (e.g., uncertainty, ambiguity) to describe the same uncertainties and the same terms to describe different uncertainties. Moreover, they fail to distinguish the sources of uncertainty (e.g., informational inconsistency, unpredictability, complexity). Regarding the *manifestations* of ‘tolerance,’ measure items assess different cognitive, emotional, and behavioral outcomes (e.g., perceptions of vulnerability, fear, indecision) and fail to distinguish between them.

Despite their substantial variability and lack of specificity, existing definitions of UT all construe uncertainty as an aversive condition that elicits negatively valenced psychological responses or outcomes, and tolerance as an endurance of these sequelae. Nicholas Carleton, developer of one the most widely used UT measures, has defined UT as a personal capacity to “endure the aversive response triggered by the absence of salient, key, or sufficient information, and sustained by the associated perception of uncertainty” [31, p. 31]. Existing definitions also treat the absence of negative psychological responses or outcomes as the defining feature or manifestation of UT: Individuals who exhibit a lower number or intensity of negative responses have greater UT, while individuals who exhibit a higher number or intensity of these responses have lower UT.

## Uncertainty tolerance as a normative concept

This prevailing conception of UT both reflects and reinforces a view of uncertainty as an aversive, undesirable condition, and UT as a psychological trait or state manifesting people’s capacity to endure this condition. A large body of empirical evidence supports this view and shows that people’s aversion uncertainty varies among individuals [6].

Strictly speaking, the prevailing conception of UT in the social science literature is a descriptive construct affirming people’s empirically observable propensity towards negative responses to uncertainty. Over time, however, this conception has taken on normative significance in various professional endeavors, becoming interpreted as not only describing how people actually respond to uncertainty but prescribing how they ideally should respond. In clinical psychology, for example, UT has become a therapeutic goal in approaches to managing anxiety disorders, based on evidence for a pathogenic role of low UT in these disorders [23, 31–33]. In medical education and practice, UT has become a proposed pedagogical and performance goal, based on emerging evidence that it improves learners’ and clinicians’ performance and well-being [2, 8, 11–13, 34, 35]. These applied endeavors reflect and reinforce the view of uncertainty as an undesirable state provoking negative responses, and UT as a desirable state marked by the absence of such responses. This normative interpretation is implicit in the very word “tolerance,” which signifies a capacity to withstand adversity, and further reinforced in the common use of the term “uncertainty tolerance” in everyday life, which reflects a broader cultural and societal aversion to risk and uncertainty [36, 37].

This normative interpretation of the prevailing concept of UT, however, poses several conceptual and empirical challenges. It fails to account for the full range and complexity of psychological responses to uncertainty. Not only do these responses vary among individuals, but uncertainty itself has multiple effects, both negative and positive. It is not only an aversive state that people avoid or endure, but also an attractive state that people seek or maintain. For example, a patient with advanced progressive cancer contemplating a promising but untested treatment may find uncertainty about the treatment’s effectiveness attractive because it offers the possibility of benefit. For this patient uncertainty may engender not fear and indecision, but hope and conviction to pursue treatment. For both patients and clinicians, furthermore, uncertainty can also draw attention to moral dilemmas that need to be contended with. Furthermore, the many cognitive, emotional, and behavioral responses uncertainty elicits are not always consistent in psychological valence; it is possible, for example, for uncertainty to elicit both fear and hope, indecision and decisiveness, and innumerable other combinations of negative and positive responses. The prevailing concept of UT, however, ignores these empirical complexities. We have thus previously proposed a more expansive, neutral descriptive definition of UT as a “*set of negative and positive psychological responses—cognitive, emotional, and behavioral—provoked by the conscious awareness of ignorance about particular aspects of the world*” [6, p. 70]. This expanded definition affirms the two-sided nature of uncertainty, and the need to account for both positively and negatively valenced outcomes in assessing UT.

The normative interpretation of the prevailing concept of UT also poses ethical challenges. The first is that the psychological valence of any given response to uncertainty does not necessarily correspond to its moral valence. Negatively valenced responses such as pessimism, fear, and indecision are not necessarily bad; conversely, positively valenced responses such as optimism, hope, and decisiveness are not necessarily good. These and other responses, in turn, may produce outcomes that may be good or bad. For example, pessimism and fear may lead a patient to forego a risky medical intervention and thereby avoid its potential harms, while optimism and hope may lead a different patient to undergo the risky intervention and thereby suffer these harms. The second, more fundamental ethical challenge lies in the use of any outcome or set of outcomes—regardless of their psychological valence—as the defining feature of UT. This consequentialist approach poses two intractable problems. The first is a demand for comprehensive knowledge of the outcomes of these outcomes; only if the various downstream thoughts, feelings, and actions elicited by a given response to uncertainty are completely accounted for can their net benefits or harms be determined. In real life, however, the number and variety of contextual factors—e.g., the nature of the clinical problem and its treatment, characteristics of the patient and situation, features of the social and healthcare environment—make it practically impossible to determine or calculate the net benefits and harms of different responses to uncertainty. The second problem is the fact that outcomes cannot be known prospectively, only retrospectively. This problem strikes to the heart of any outcomes-focused conception of UT given that the unknowability of future outcomes is precisely the problem that demands “tolerance” to begin with. The outcomes of any given response to uncertainty are themselves uncertain.

Making outcomes the normative criterion for evaluating UT thus does not resolve but merely recapitulates this problem because it presupposes the very knowledge that people do not and cannot have. At best, any outcomes-focused conception of UT can only be a standard for retrospectively evaluating people’s past responses to uncertainty, not for prospectively guiding their current responses. It can help people learn from their experiences, but cannot provide definitive answers about how to respond to uncertainty in real time.

## Rethinking uncertainty tolerance: from endurance to adaptation

The prevailing normative conception of UT as the capacity to endure aversive effects of uncertainty, manifest by the absence of negatively valenced psychological responses, is thus deficient as a normative ideal. To be practically useful, a normative conception of UT must be more than a standard for evaluating—incompletely and after the fact—people’s responses to uncertainty. It must be a guiding ideal that enables clinicians and patients to actually live with uncertainty—to think, feel, and act in spite and because of their lack of knowledge of future consequences.

A coherent and practically useful normative conception of UT must also accommodate the fundamentally ambiguous and complex nature of uncertainty and its outcomes. Uncertainty—the object of UT—is not just aversive; it produces a wide range of outcomes that are both negative and positive from a psychological standpoint and good and bad from a moral standpoint, depending on the individual and circumstance. Correspondingly, tolerance—the defining feature or manifestation of UT—entails more than the endurance of outcomes that are negative or bad; it also entails the enjoyment of outcomes that are positive or good, and the balancing of these and other responses. As a normative ideal, UT thus cannot be defined solely in terms of the extent to which a given individual finds uncertainty aversive or attractive, exhibits any particular response to uncertainty, or experiences any given outcome. It must be defined in a way that acknowledges the absence of definitive answers to the question of how to respond to uncertainty, and changes the goal of managing uncertainty from ‘getting it right’—that is, achieving correct outcomes as defined by existing norms—to adapting to the absence of such norms and our inability to know what is right.

Philosopher Georges Canguilhem offered useful insights about the nature of this adaptation process in his classic work, *The Normal and the Pathological* [38]. Canguilhem construed adaptation broadly as a process of adjusting to changing environmental conditions, and viewed this process as the fundamental basis of human health. His key insight for our purposes was his more specific interpretation of adaptation as a “normative capacity” consisting of an ability to rise above current norms of functioning, defined by the “imposed circumstances” of one’s environment, to create and establish “new norms in new situations” that maintain or improve health in changing conditions [38]. This insight led Canguilhem to define health itself as a “margin of tolerance for the inconstancies of the environment” [38, p. 197]; the broader the margin, the greater the ability not only to follow but to rise above existing norms of functioning to create new, adaptive norms, and the greater one’s level of health. Canguilhem thus argued that health entails being “not only possessor or bearer but also, if necessary, creator of value, establisher of vital norms” [38 p. 201].

We believe this understanding of adaptation provides a useful way of thinking about UT, which we similarly construe as a margin of tolerance but specifically for the knowledge deficits that define people’s current norms of functioning. Following Canguilhem, we construe the adaptation process at the core of UT as a normative capacity—an ability to continuously create and establish new individual- and situation-specific norms that define how to manage uncertainty and what outcomes to pursue in changing circumstances. We believe it is this creative normative capacity that enables people to adjust to their inability to know how to move forward, and represents a coherent foundation for a normative conception of UT. Unlike endurance, adaptation accommodates the unknowability of the future and the morally ambiguous nature of uncertainty and its effects, both of which render it impossible to determine whether any given response to uncertainty is right. It acknowledges the context-dependency of all responses to uncertainty, and leaves room for a broader range of responses beyond simply enduring its negative effects.

The practical challenge of an adaptation-based conception of UT, however, is that it assigns no inherent moral value to any particular response to or outcome of uncertainty, and thus offers no clear, concrete goal for efforts to manage uncertainty. Furthermore, adaptation can be valued in different ways—either instrumentally, as a means of achieving some desired end, or intrinsically, as an end in itself. In healthcare, for example, adaptation could simply be valued as a means of improving patient or clinician well-being. Such an instrumental view is compelling given the paramount importance of outcomes in healthcare, but poses the same problems of all consequentialist approaches to UT. In requiring prospective knowledge of future outcomes, it simply begs the question of how to deal with the absence of such knowledge. To be practically useful, an adaptation-based conception of UT needs to reflect and reinforce the value of adaptation as an end in itself for clinicians and patients; it must encourage them to constantly adjust their responses to uncertainty—to create and enact new individual- and situation-specific responses—*regardless of the outcomes*. This paradoxical disregard of outcomes is necessary not only in theory, to address the epistemological and ethical problems posed by a consequentialist approach to UT. It is also necessary in practice, to address the fundamental existential problem posed by uncertainty: how to move forward in spite of the unknowability of what lies ahead. Addressing this problem requires a capacity to eventually detach oneself from desired outcomes; to forget about what-ifs and act no matter what the desired consequences may be; to take a leap of faith. The object of this faith, furthermore, is the power of adaptation itself. What ultimately enables clinicians, patients, and all human beings confronting uncertainty to look beyond all possible outcomes and to step into the unknown future is a positive conviction that they will always be able to adjust to whatever the future might bring.

One could not move forward in the face of uncertainty without this conviction. The patient with advanced cancer could not make a final choice to accept or reject a treatment option with unknown benefits and harms without the ability to eventually stop contemplating what is possible but unknowable, to act based on norms she creates in the context of her own unique circumstances, experiences, life story, and values, and to accept whatever outcomes may befall her. This self-affirming faith in the creative power of adaptation is the defining feature of UT, the foundational existential capacity that enables individuals to look beyond—and rise above—the predicted, desired, and undesired outcomes of their chosen responses to uncertainty, and that renders their uncertainty about these responses tolerable. Adaptation is the *telos*, the guiding goal of efforts to manage uncertainty, and the basis for a normative conception of UT [18].

Integrating all these insights, we offer the following working definition of UT as a normative ideal: *The adaptive capacity to create and enact individual and contextual responses to uncertainty*. This definition construes UT as a capacity for adaptation rather than endurance, and makes outcomes a secondary focus of people’s efforts to manage uncertainty. It makes the primary focus of UT the adaptation process itself, which empowers people to move forward regardless of what might happen and to live with the consequences. The term “creating and enacting” highlights the active, intentional, dynamic nature of UT; it involves the constant, never-ending generation of new responses to uncertainty. The terms “individual and contextual” highlight the normative nature of UT; it is a capacity, as Canguilhem argued, to create and establish new, individual- and situation-appropriate norms to guide one’s responses to uncertainty. At the same time, it acknowledges how the response to uncertainty is context-dependent; there is no single right response for all individuals and situations. The normativity of UT lies in the capacity not to simply endure, but to set new norms by adapting to the context.

Importantly, this adaptation-based conception does not render the outcomes of people’s efforts to manage uncertainty irrelevant to UT, nor does it entail completely disregarding efforts to improve patient or clinician well-being. An adaptation-based normative conception of UT simply provides a complementary perspective that acknowledges the possibility that these and other outcomes may not be realized, and the necessity of somehow preparing for this possibility. It expands the goals of managing uncertainty beyond achieving desirable outcomes to building clinicians’ and patients’ capacity to constantly adapt, regardless of the outcomes.

## Rethinking uncertainty tolerance: from outcomes to virtues

An adaptation-based conception of UT suggests the need for a shift in focus in our efforts to manage uncertainty: from outcomes to the human capacities that enable them, from the important but unanswerable questions of *what will happen* and *what to do* to the equally important but answerable question of *how to be*. Philosophers since the time of Aristotle have answered this question using the concept of moral virtues—excellent character traits or personal dispositions, such as honesty and courage. Virtues manifest not only excellence and rightness in not only what one does, but in how one fundamentally is as a person. They represent what social psychologists Charles Carver and Michael Scheier have called “be goals”—abstract principles of living or ways of being that represent ultimate ends of human life and aspects of one’s self-concept [39–41]. They constitute the central focus of a major ethical theory that regards virtues as the primary guiding principle for living and judging one’s actions [42], over and above other criteria such as the consequences of one’s actions (consequentialism) or the duties or rules that should guide actions (deontology). Most importantly for our purposes, virtues represent personal capacities that may foster the higher-order capacity to adapt to uncertainty, and thus provide a useful focus for a normative conception of UT. Philosopher Alasdair MacIntyre characterized the “ability to live with uncertainty” as itself a virtue of paramount importance for modern life [43]. Psychologist Robert McGrath further argued that the ultimate function of virtues is to satisfy fundamental human needs that promote adaptation [44], of which the need “to reduce uncertainty about the environment” is the most fundamental of all [45].

We believe virtues help one rethink UT and move from an endurance-based to an adaptation-based normative conception. They represent a missing link in the prevailing conception of UT; they fill the moral gap left that remains after unseating outcomes as the sole criterion for evaluating people’s responses to uncertainty. Virtues address the core problem of uncertainty—the unknowability of future outcomes and the impossibility of prospectively determining whether any given response to uncertainty is right—and thus provide a more coherent, meaningful goal and focus of efforts to manage uncertainty. They represent the constituents of UT—fundamental human capacities that ultimately enable people to adapt to uncertainty. Unlike future outcomes, virtues are knowable and accessible qualities that provide an actionable focus of efforts to manage uncertainty; they lie within the grasp of every individual and can be cultivated. When what will happen and what to do are unknown, virtues specify how to be. They specify ideal ways of being—as opposed to states of being—that individuals can actively and intentionally cultivate as they strive to move forward into the unknown.

The important question is, what specific virtues foster adaptation to uncertainty and represent the critical elements of a normative conception of UT? The potential list is long and encompasses what virtue ethicists have classified as moral virtues (e.g., honesty, temperance), which encompass qualities or habits of being that promote human flourishing and goodness, and intellectual or epistemic virtues (e.g., curiosity, open-mindedness), which encompass qualities or habits of thinking that promote the acquisition and appropriate use of knowledge. To these categories McGrath has added, based on empirical evidence from virtue measurement studies, a third key domain of “self-regulatory virtues” (e.g., temperance, conscientiousness) which promote effective behavioral self-control “in the service of goal achievement” [44, 45]. Positive psychologists Christopher Peterson and Martin Seligman have developed a well-known hierarchical taxonomy of virtues, at the top of which are six core virtues identified by philosophers: wisdom, courage, humanity, justice, temperance, and transcendence [46]. At the next, lower level are twenty-four lower-level virtues that Peterson and Seligman designate as “character strengths”—psychological processes or mechanisms that correspond to these virtues and define specific ways of enabling them [46]. For example, the character strengths of creativity and curiosity enable the virtue of wisdom. Character strengths thus represent instrumental virtues that enable the expression of higher-level, intrinsic virtues.

In theory, all of these virtues—both intrinsic and instrumental, as well as moral, intellectual (epistemic), and self-regulatory—may help people adapt to uncertainty and represent critical elements of UT. In practice, however, several problems weigh against simply including the entire range of virtues within a normative conception of UT. First, not all virtues are equally important in dealing with any given problem; psychologist Barry Schwartz and political scientist Kenneth Sharpe have called this the “problem of relevance” [47]. Uncertainty differs from other problems of human life, and uncertainty in healthcare differs from uncertainty in other contexts. Uncertainty is a distinct metacognitive state that arises from specific sources, pertains to specific issues, and has specific psychological effects that influence the well-being of patients, clinicians, and other persons [48]. To be relevant to UT, any given virtue must somehow address these specific aspects of uncertainty and its effects. A second problem is the “problem of specificity,” the fact that any given virtue can also suggest many different responses to the problem [47]. For example, courage may suggest both enacting and foregoing a risky medical intervention, depending on what possible consequences are most feared. Related to this is the “problem of conflict,” which arises when different virtues suggest incompatible responses [47]. For example, honesty and kindness may suggest opposing approaches in a physician’s discussion of prognosis with an advanced cancer patient (e.g., disclosing vs. withholding information). A final problem is the paradox that at high levels of expression virtues can also be bad or morally undesirable depending on the situation; for example, high levels of courage could discourage a patient from life-saving treatment. Psychologists Vincent Ng and Louis Tay have called this the “too-much-of-a-good-thing” problem, although they reference Aristotle’s doctrine of the “golden mean” in arguing that properly understood, it is not possible to have “too much” virtue; by their very nature, virtues imply “situation-specific optimality” in their level of expression [49]. Courage, for example, by definition lies at a situation-specific, intermediate space between extreme vice states of foolhardiness and cowardice. Nevertheless, this problem suggests that virtues are inherently quantitative as well as qualitative, with varying levels of expression in different situations [44, 49, 50].

These many problems challenge any effort to identify virtues that represent the most critical elements of a normative conception of UT. Although all virtues may play at least some role in helping people adapt to at least some uncertainties in at least some situations, they differ in relevance and specificity and have conflicting effects and varying levels of expression. Furthermore, a conception of UT that simply included the entire body of identified virtues would be impractical as a normative ideal; it would require clinicians and patients to attend to and cultivate numerous virtues with minimal relevance to a situation. A practically useful virtue-focused normative conception of UT, therefore, must strike a reasonable balance between comprehensiveness and parsimony: it must be sufficiently broad to include virtues that are relevant to most situations, yet sufficiently narrow to be actionable. Achieving this balance requires identifying virtues that most directly address the unique adaptational challenges uncertainty raises in the healthcare context, and accepting that any subset is necessarily incomplete, imprecise, and provisional.

## Uncertainty‑tolerant virtues in healthcare: insights from existing frameworks

We will now attempt to develop a reasonably balanced, preliminary list of uncertainty-tolerant virtues in healthcare that can inform future work on this problem. Virtue ethicists, virtue epistemologists, and psychologists have developed numerous frameworks that vary in their content, comprehensiveness, and goals; most have focused broadly on virtues in human life, although some have focused specifically on healthcare and related endeavors (e.g., science, engineering) [44, 46, 50]. Given our focus on UT in healthcare and the purposefully preliminary nature of our effort, we draw on insights from a selective rather than comprehensive review of frameworks developed for healthcare-related fields.

A useful framework for this purpose is the “taxonomy of technomoral virtues” put forth by philosopher Shannon Vallor, aimed at helping people deal with new and emerging technologies [51]. Vallor identifies twelve virtues as most critical in dealing with uncertainties raised by these technologies: honesty, self-control, humility, justice, courage, empathy, care, civility, flexibility, perspective, magnanimity, and technomoral wisdom. Vallor argues that “technomoral humility” enables individuals to achieve an adaptive balance between excessive “techno-optimism” and excessive “techno-pessimism” in the face of uncertainty about the true value of a novel technology. Moreover, “technomoral courage” enables individuals to achieve an adaptive balance between excessive hope and excessive fear in the face of uncertainty about value of a novel, potentially beneficial technology [51]. Finally, “technomoral flexibility” enables individuals to achieve an adaptive balance between excessive accommodation and excessive disregard of divergent viewpoints in the face of uncertainty about the norms and values that should guide responses to a new technology.

In the domain of engineering, ethicists have advocated for virtue-based over consequentialist and deontological approaches to decision making, and put forth several taxonomies of virtues that help people deal with risk and uncertainty in engineering practice [52–56]. For example, Bergen and Robaey developed a taxonomy that includes not only general moral and intellectual virtues that are broadly applicable in daily life, but several “specifically engineering virtues”—e.g., creativity, openness to criticism, sensitivity to risk, respect for nature, commitment to the public good—that enable engineers to deal with uncertainties arising from various sources, including limitations in scientific knowledge and conflicts in values and norms [56].

In the domain of healthcare, scholars have identified numerous virtues with special relevance to clinical practice [57–59]. Pelligrino and Thomasma, in their seminal work *The Virtues in Medical Practice*, developed a framework of eight foundational virtues: *phronesis*, fidelity to trust, compassion, justice, fortitude, temperance, integrity, and self-effacement [60]. Pelligrino and Thomasma argue that this set of virtues, like those put forth by Vallor and engineering ethicists, serve a variety of ethical functions and are not justified exclusively by their value in helping people tolerate uncertainty; however, they highlight the critical roles that specific virtues play in this endeavor. The primary example is *phronesis*, or practical wisdom—which Aristotle considered a master virtue that allows people to discern and exercise the specific virtues needed to achieve *eudaimonia*, or human flourishing. *Phronesis* serves numerous regulatory functions [61, 62], but Pelligrino and Thomasma highlight its importance in enabling clinicians to confront uncertainties about the appropriate goals of treatment and the appropriate actions to be taken to achieve these goals—uncertainties that cannot be completely eliminated due to fundamental limitations in human knowledge. Pelligrino and Thomasma also acknowledge how the virtues of temperance, integrity, justice, fortitude, and self-effacement empower clinicians in different ways to regulate their own responses to uncertainty—e.g., to avoid extreme impulsiveness, unreliability, arbitrariness, resignation, and excessive risk taking—while fidelity to trust and compassion each manifest the uniquely altruistic function of medicine and help clinicians to help their patients bear the burden of the uncertainties at hand [60]. All eight virtues identified by Pelligrino and Thomasma can thus be construed as promoting UT, even though they are broader in their focus, functions, and ethical importance [63].

Specific to the problem of UT in healthcare are two more recent frameworks. Reis-Dennis and colleagues’ framework consists of three virtues construed as serving a “corrective” function—to “help physicians avoid the specific uncertainty-related pitfalls” or tendencies towards “fight or flight” responses that may not be appropriate to individual clinical situations: (1) *courage* “corrects for the tendency to ‘flee’ from uncertainty too hastily”; (2) *diligence* “mitigates the impulse to give into it too easily”; and 3) *curiosity* “inspires practitioners of all tolerance levels to confront it productively” through “engagement with medical and scientific scholarship” [17, p. 2410]. This view of the functions of uncertainty thus reflects and reinforces the prevailing conception of uncertainty as a fundamentally aversive phenomenon that needs to be endured, and tolerance as a matter of minimizing negative responses to it. However, the authors also acknowledge the moral ambiguity of both negative and positive responses to uncertainty in arguing that virtues enable physicians to avoid the “extremes of tolerance and intolerance for uncertainty” [17, p. 2409]. In this view virtues are thus extraneous to UT; they comprise a separate regulatory capacity to mitigate both uncertainty tolerance and intolerance (manifested, respectively, by negatively and positively valenced responses to uncertainty).

Another healthcare-specific, UT-focused virtue framework put forth by one of us (Han) identifies three specific virtues—humility, flexibility, and courage—which it treats as constitutive rather than corrective of UT, integral components of an overarching capacity for adaptation [18]. In this framework the function of virtues is not to either avert negative uncertainty responses or promote positive ones, or to compensate for individuals’ tendencies towards one type of response or another. It is to promote adaptation to uncertainty—an intentional self-regulatory process that entails acts of transcendence in three metaphorical, existential directions, which different virtues enable. *Humility* enables individuals to transcend uncertainty in a vertical direction—to acknowledge and rise above their uncertainty (their metacognitive awareness of their ignorance [48]), to achieve an even higher-order presence of mind that allows them to accept and manage their uncertainty in a deliberative manner. *Flexibility* enables individuals to transcend their uncertainty in a horizontal direction—to move across their varied psychological responses to uncertainty and to couple and uncouple, combine and recombine them in ways that allow them to adapt to the situation at hand. *Courage* enables individuals to transcend their uncertainty in a forward direction—to move ahead into the unknown and unknowable future, to take action in spite of their uncertainty. These virtues are thus constitutive of UT—essential elements of the higher-order capacity to adapt to uncertainty. This view of UT and its relationship to key virtues aligns with Canguilhem’s positive conception of adaptation as a normative capacity manifesting more elemental capacities of transcendence (the capacity to rise above one’s current self and circumstances), creativity (the capacity to generate new norms of living), and self-determination (the capacity to realize one’s goals) [38, 64]. Humility, flexibility, and courage map to these elemental capacities, and enable individuals to maximize their ‘margin of tolerance’ of the uncertainties at hand.

## An integrative taxonomy of uncertainty-tolerant virtues

Existing virtue frameworks in healthcare and related fields vary in content and comprehensiveness, but suggest that several virtues may enhance clinicians’ and patients’ capacity to adapt to uncertainty and thus represent key elements of UT. These frameworks also offer useful insights on how individual virtues promote UT. For example, Vallor’s interpretation of courage as helping individuals achieve an adaptive balance not between cowardice and foolhardiness (as Aristotle envisaged [65]), but between fear and hope, highlights a distinctive role of courage in UT. Existing frameworks also manifest differing conceptions of the nature and effects of uncertainty, the goals of managing uncertainty, and the meaning of UT. Most frameworks construe uncertainty as an aversive phenomenon that needs to be endured and tolerance as the absence of negative responses, although some frameworks affirm moral ambiguity in the nature of uncertainty and one’s responses to it. In our view, Han’s framework affirms this ambiguity most explicitly in identifying adaptation—rather than the achievement of any given outcome—as the ultimate goal of managing and uncertainty and the defining feature of UT. In this adaptation-based view, it does not make sense to talk about being excessively tolerant or intolerant of uncertainty, just as it does not make sense—from an Aristotelian perspective—to talk about being excessively virtuous [49]. UT entails an adaptive, situationally optimal combination and level of responses to uncertainty—regardless of their psychological valence. In this view, virtues represent essential, fundamental elements of UT; they are constitutive rather than ‘corrective’ of UT.

In the final analysis, the “truth” of any given conceptual taxonomy depends on its usefulness in achieving particular goals [44, 50, 51], and existing taxonomies may all be useful in identifying guiding ideals for clinicians’ and patients’ efforts to manage uncertainty in healthcare. The question remains, however, of what specific combination of virtues is most useful. Acknowledging that there is no single right answer to this question and currently no empirical evidence on the usefulness of any virtue framework in helping clinicians and patients manage uncertainty, we integrate insights from existing frameworks to propose a provisional, parsimonious taxonomy of uncertainty-tolerant virtues in healthcare. We justify our selection of virtues by demonstrating their value in addressing fundamental adaptational challenges posed by uncertainty in healthcare.

**Fig. 1:**
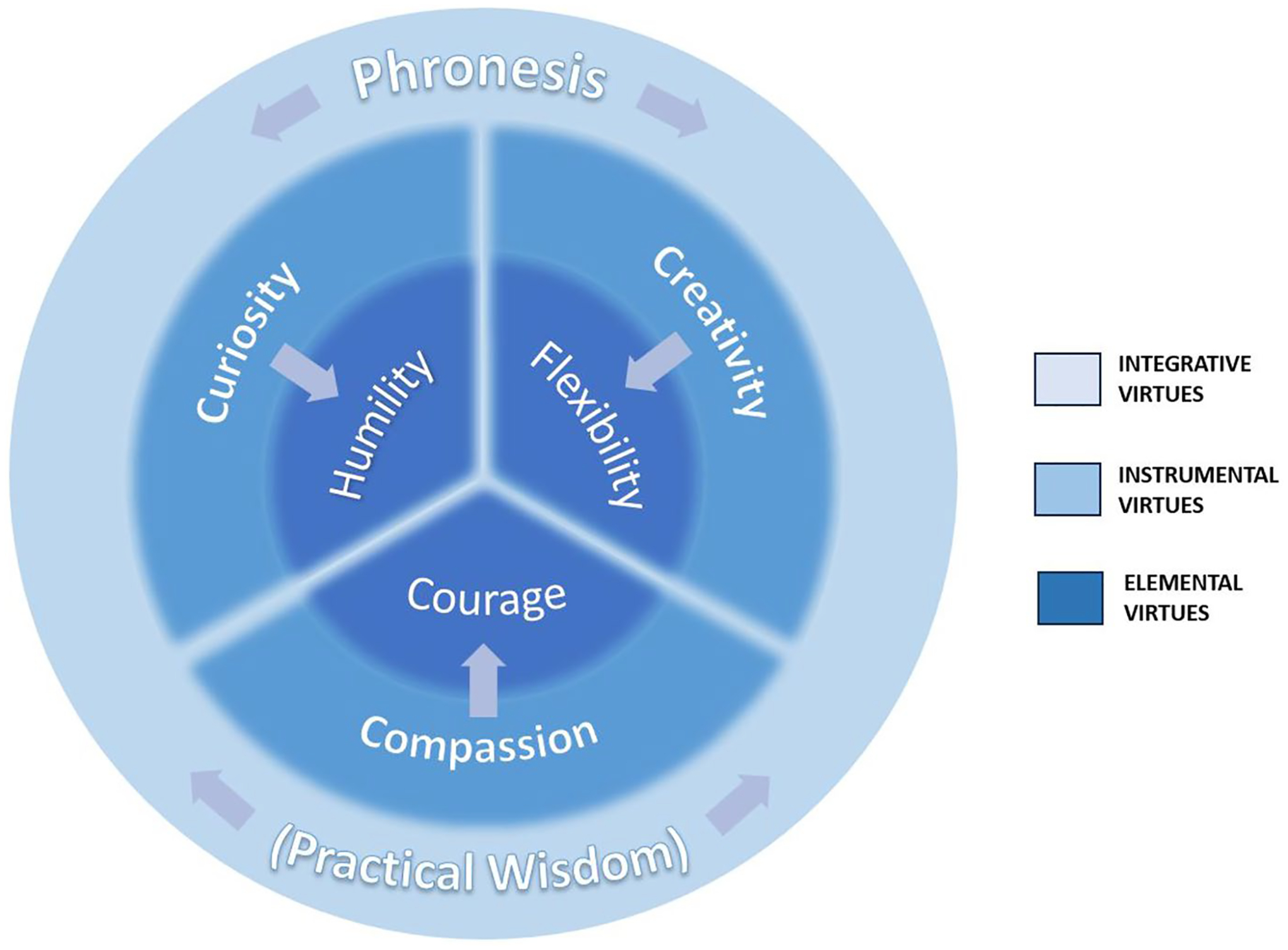
Integrative taxonomy of uncertainty-tolerant virtues

Our integrative, hierarchical taxonomy distinguishes between constituent virtues residing at three conceptual levels: (1) primary, consisting of *elemental virtues* that enable key tasks of dealing with uncertainty; (2) secondary, consisting of *instrumental virtues* that foster the intrinsic virtues; and (3) tertiary, consisting of *integrative virtues* that foster the appropriate application of both the intrinsic and instrumental virtues to specific situations in life in general and medicine in particular (Fig. 1). We do not further distinguish individual virtues as either moral, intellectual, or self-regulatory; we believe these functional categories are not mutually exclusive [56].

At the primary, elemental level are the three virtues that Han previously proposed as core capacities of UT: *humility*, *flexibility*, and *courage* [18]. These virtues serve multiple functions beyond enabling UT; however, we single them out as core capacities for UT because of the unique existential needs that uncertainty raises, detailed previously. Importantly, we construe these elemental virtues as not only moral, intellectual, and self-regulatory [66, 67], but also existential in nature; the acts of transcendence that they enable represent the most fundamental endeavors of human life. Philosopher and theologian Paul Tillich, in his classic work, *The Courage To Be*, viewed courage as a fundamental capacity “rooted in the whole breadth of human existence and ultimately in the structure of being itself” [68, p. 1]. It is not only a moral or intellectual capacity, but an essential power of “self-affirmation ‘in-spite-of,’ that is in spite of that which tends to prevent the self from affirming itself” that enables individuals to move forward in the face of the threat of nonbeing, the ultimate unknown [68, p. 41]. We view humility and flexibility in the same way, as existential capacities that enable one to not only act and think properly, but to live with uncertainty. Each virtue helps one to transcend uncertainty in different ways—humility by helping one recognize the limitations of one’s knowledge, flexibility by helping one recognize the limitations of one’s different possible responses, courage by helping one recognize the limitations of one’s control over one’s future fates. In the very recognition of such existential limitations, Tillich argued, one rises above them; the recognition itself is a transcendent act [68].

Humility helps both our exemplary patient with advanced cancer and her clinician to acknowledge what is known, unknown, and unknowable about the benefits and harms of all treatment options and the prognosis of all patients, which reflect fundamental limitations in not only the adequacy of all empirical evidence but its applicability to any individual case [69]. Humility enables both parties to affirm that there is no single, pre-determined right answer about what will happen; therefore, they must do the best they can with the knowledge they have. Humility thus empowers both the patient and clinician to transcend their uncertainty about the future; it liberates them to see uncertainty as not only closing down but opening up possibilities, expanding the potential space of different outcomes. It unburdens both parties, as we have previously argued, from the “tyranny of unrealistic expectations about the existence of singular, definitive answers and our ability to find them” [70, p. 574].

Flexibility helps the patient and her clinician to acknowledge the finite range of potential responses to uncertainty—cognitive, emotional, behavioral—which ultimately include either accepting or rejecting, enacting or foregoing the treatment at hand. Flexibility enables both parties to affirm that there is no single, pre-determined right answer about what to do; rather, they must create their own right answers that fit the uncertainty, the patient, and the situation. Flexibility thus empowers both the patient and clinician to transcend their uncertainty about what to do; it frees them to consider and enact different responses, even in apparently inconsistent combinations (e.g., fear and action, confidence and inaction), or to change their minds—to alter their responses—as available information or clinical circumstances evolve (e.g., first refusing then accepting treatment, or pursuing alternative courses of action). It unburdens both parties from the stifling expectation of a one-size-fits-all approach to uncertainty, and the anticipatory regret and guilt that can prevent them from considering all options.

Finally, courage helps the patient and her clinician to acknowledge the paradoxically limited relevance of both their desired and expected outcomes on the task at hand. Courage enables both parties to affirm that they lack complete control over these outcomes; therefore, they must ultimately disengage from these outcomes and act. Courage thus empowers both the patient and clinician to transcend their uncertainty about whether to act on a given clinical option; it allows them to let go of their desires and expectations and to move forward regardless of the outcomes, with faith in their ability to deal with whatever their eventual fate may be. It unburdens both parties from the unrealistic expectation of control over the future, and the necessary but paralyzing desire for a good outcome.

At the secondary, instrumental level are three virtues that serve the adaptive function of fostering the elemental capacities of humility, flexibility, and courage. *Curiosity* manifests and promotes openness to the acquisition of new information that may either reduce one’s ignorance or clarify the extent of it, and thereby fosters humility. It embodies the basic inquisitiveness and need for knowledge inherent to all efforts to explore the environment [45]. It motivates the patient and clinician to learn more about the clinical problem, the available treatment options and their potential outcomes, and the patient’s values. *Creativity* manifests and promotes imagination and the generation of new insights and solutions to one’s lack of knowledge and how to manage it, and thereby fosters flexibility. It embodies the basic ability to let go of old norms and to establish new norms inherent to all efforts to adjust to the environment. It motivates the patient and clinician to come up with a treatment plan that works for them—regardless of whether it aligns with norms outlined in clinical practice guidelines or treatment algorithms. *Compassion* manifests and promotes human concern, caring, and empathy—which may be directed towards not only other persons but oneself—in the face of one’s uncertainty and one’s responses to it, and thereby fosters courage. It embodies the basic power of self-affirmation inherent to all efforts to deal with threats in life. Compassion is also critically important in healthcare given the fundamentally caring nature of this endeavor; however, it plays a specific, special role in UT in promoting the courage that both clinicians and patients need to move forward into the unknown. It motivates both parties to understand and forgive each other—and themselves—for their limited knowledge and control over the future, and for whatever outcomes they may eventually experience.

We propose this mapping between primary and secondary virtues loosely; curiosity, creativity, and compassion may each play a role in fostering any of other three primary virtues. The fuzzy borders in Fig. 1 are meant to represent their indistinct, fluid, conceptual and functional boundaries. Furthermore, virtues may not always operate in a hierarchical or unidirectional fashion; instrumental virtues may promote UT directly, while elemental virtues may promote UT indirectly by promoting instrumental virtues. Nevertheless, we believe this mapping reflects strong functional affinities between particular virtues. We also believe that numerous other virtues operate at an instrumental level to support the elemental virtues. These include virtues identified in previously mentioned frameworks—e.g., honesty, justice, magnanimity—all of which may also foster humility, flexibility, and courage in particular situations of uncertainty. However, we single out curiosity, creativity, and compassion as having particularly important roles across different healthcare situations.

At the tertiary, integrative level are virtues that serve the executive function of fostering the appropriate application of intrinsic and instrumental virtues. The most important of these is *phronesis*, or practical wisdom—which in our framework represents a higher-order, meta-virtue that enables individuals to discern what specific elemental and instrumental virtues are needed to adapt to uncertainty, and how these virtues should be integrated and exercised. Other self-regulatory virtues may also enable individuals to enact elemental and instrumental virtues that help them adapt to uncertainty [45]; examples include not only equanimity, which can help individuals balance conflicting responses to uncertainty, and diligence [17], which can help individuals persevere in these efforts. *Phronesis*, however, represents the capacity to regulate even these virtues, and is thus the quintessential virtue of UT—the overarching regulatory capacity that ultimately enables people to adapt to uncertainty. It allows both the patient and clinician to know what degree of humility, flexibility, courage to exercise at any given moment, and which other virtues may also need to be cultivated to adapt to the uncertainties at hand.

## Towards a normative conception of UT: promises, challenges, and future needs

As an alternative to the prevailing endurance-based, outcomes-focused normative conception of UT, we have put forth a new conception that construes adaptation as the ultimate goal of efforts to manage uncertainty, UT as the personal capacity to adapt to uncertainty, and virtues as the essential components of this capacity. As the foundation for this new conception, we have developed a provisional taxonomy of uncertainty-tolerant virtues that is purposefully not comprehensive but theoretically coherent and practically useful, and aimed at providing clinicians and patients with a finite, manageable set of guiding ideals applicable to most uncertainties and situations. We believe that several key features of this integrative model make it a promising normative ideal for efforts to manage uncertainty in healthcare.

1. *Normalizing uncertainty*. The model reinforces a view of uncertainty as a normal, expected experience that should be accommodated rather than eliminated. It does not deny its negative effects, but rather affirms its potentially positive effects and the morally ambiguous, context-dependent nature of all of them. It thus makes UT a matter of not simply enduring uncertainty but adapting to it—achieving a balance of effects that is optimal to the individual and situation, which may be both negative and positive. It thus promotes a more holistic, agnostic, integrative perspective on uncertainty and the goals of managing it, and more realistic epistemic expectations that that may themselves make uncertainty less aversive, more acceptable, and even sometimes desirable to both clinicians and patients [71–75].
2. *Building capacity*. The model makes building capacity—i.e., cultivating personal character strengths that foster our ability to adapt to uncertainty—the key task of managing uncertainty. It does not supplant the critical task of seeking knowledge, but rather supplements it with the equally critical task of adapting to our uncertainty when what will happen and what to do cannot be resolved by existing knowledge. Our model offers an alternative focus for our attention and action: how to *be*. It redirects one’s gaze and actions inward, to what one can change in oneself to better manage uncertainty.
3. *Transcending outcomes*. The model makes transcending outcomes a core goal of managing uncertainty. It does not diminish the importance of improving outcomes such as health and well-being, but rather acknowledges the equal, existential importance of coping with the fundamental unknowability and uncontrollability of these outcomes. It affirms how achieving this goal paradoxically requires looking beyond and detaching from outcomes—letting go of one’s hopes and expectations about what might happen in order to move forward into the unknown, with faith in the power of adaptation itself.

In these ways, our new normative model of UT promotes a particular mindset, approach, and management goal that could help clinicians and patients engage with uncertainty more intentionally, broaden their repertoire of strategies to adapt to it, and address its core existential challenges more directly. We believe the model could help both parties visualize, reflect upon, and cultivate their own character strengths, and thereby take a more intentional approach to managing different uncertainties.

We also believe the model’s relevance to healthcare extends beyond UT. Its component virtues undoubtedly serve functions beyond the management of uncertainty per se, and promote sound clinical practice in other ways. By the same token, many other virtues not included in our framework may promote UT. However, we believe that the lack of specificity in the function of individual virtues does not undermine the theoretical validity and practical utility of highlighting particular virtues with special relevance to UT; it simply requires a flexible approach to the task.

Moving forward, more research—conceptual and empirical, theoretical and applied—is needed to refine the model and assess its value in helping clinicians and patients manage uncertainty. Conceptual research, bridging the disciplines of virtue ethics, virtue epistemology, positive psychology, and clinical medicine, is needed to identify the most useful virtues to include in the model. Empirical research is needed to develop valid and reliable measures of virtues [44, 49, 50, 76] as well as the adaptive capacity that constitutes UT. This is a critical challenge given the inherent context-dependency of this construct; no outcomes are clearly indicative of ‘good’ adaptation for all individuals and situations. One useful approach may be to measure uncertainty-tolerant virtues themselves (e.g., humility, flexibility, courage) together with cognitive, emotional, or behavioral manifestations of these virtues that would be expected to help people adapt to uncertainty—e.g., “mature” epistemological beliefs that acknowledge the incomplete, provisional, and pluralistic nature of knowledge [63, 77, 78]; “critical openness”—a receptiveness to learning new ideas, evaluating them critically, and changing one’s views in light of evidence [79]; and the capacity to let go or detach from outcomes [80–82]. These measurement efforts could ultimately help answer the critical question of how different virtues affect clinicians’ and patients’ management of uncertainty, as well as desired outcomes such as well-being. Emerging evidence suggests that virtues may influence clinician well-being [63], but more research is needed to confirm these findings and evaluate how virtues specifically affect how both clinicians and patients manage uncertainty, and to examine how these effects might be moderated by contextual factors. For example, particular uncertainty-tolerant virtues may have different effects for a healthy person facing uncertainty about the meaning of an unexplained symptom or an abnormal cancer screening test result, than for a person with advanced-stage cancer facing uncertainty about her prognosis or the value of pursuing a risky anticancer treatment.

Finally, applied research is needed to understand how to effectively cultivate virtues among clinicians and patients. Past efforts to develop and implement virtue-cultivating interventions in educational settings outside of healthcare are a promising foundation for such research [66, 76, 83], along with similar efforts in medical education aimed at promoting professional identity formation [84–86]. Fraser and Greenhalgh have argued that complexity in medicine and healthcare demands a paradigm shift in the goals of medical education, from competence to “capability”—the “ability to adapt to change, generate new knowledge, and continuously improve performance” [87, p. 799]. We view uncertainty as the underlying problem raised by complexity, and virtues as key components of the capability that needs to be fostered. More work is needed to develop and implement virtue-cultivating interventions in medical practice—a formidable task given the numerous well-known barriers faced by all efforts to improve healthcare quality, including time constraints and existing structures and processes of care. However, an equally important barrier is inherent to the new conception of UT. As opposed to the prevailing conception that views uncertainty simply as an aversive state and UT as a matter of enduring it, the new model poses an agnostic, integrative view of uncertainty as having no single necessary moral valence, mandating no universal response or outcome, and requiring individuals to create their own unique solutions to the problem. This pluralistic, particularistic, and constructivist perspective not only embodies but also *demands* some level of UT to accept. It raises the need for strategies that can help clinicians and patients not only cultivate uncertainty-tolerant virtues, but adopt a new way of thinking about uncertainty and its place in their lives.

In the final analysis, adopting this new normative model of UT—like the task of managing uncertainty itself—is a matter of faith: it requires a will to believe in a capacity rather than an outcome, a potentiality rather than a reality. It goes against the grain of usual approaches to uncertainty in medical practice and education and perhaps everyday life, and may not be compelling for anyone who does not already believe that the usual approaches to uncertainty are inadequate, and that a different guiding ideal is needed. If one sees any truth in this belief, however, then the model could be a fruitful starting point for an alternative approach, and in this spirit we put it forward with humility, flexibility, and courage.

## References

[1] Uncertainty in medicine. 2010. Lancet 375 (9727): 1666. 20480939

[2] Simpkin AL, and Schwartzstein RM. 2016. Tolerating Uncertainty - The Next Medical Revolution? New England Journal of Medicine 375 (18): 1713–1715. 10.1056/NEJMp1606402 27806221

[3] Gerrity MS, DeVellis RF, and Earp JA. 1990. Physicians’ reactions to uncertainty in patient care. A new measure and new insights. Medical Care 28 (8): 724–736. 10.1097/00005650-199008000-00005 2385142

[4] Geller G, Faden RR, and Levine DM. 1990. Tolerance for ambiguity among medical students: Implications for their selection, training and practice. Social Science & Medicine 31 (5): 619–624. 10.1016/0277-9536(90)90098-d 2218644

[5] Geller G, Tambor ES, Chase GA, and Holtzman NA. 1993. Measuring physicians’ tolerance for ambiguity and its relationship to their reported practices regarding genetic testing. Medical Care 31 (11): 989–1001. 10.1097/00005650-199311000-00002 8231339

[6] Hillen MA, Gutheil CM, Strout TD, Smets EMA, and Han PKJ. 2017. Tolerance of uncertainty: Conceptual analysis, integrative model, and implications for healthcare. Social Science & Medicine 180:62–75. 10.1016/j.socscimed.2017.03.024 28324792

[7] Strout TD, Hillen M, Gutheil C, Anderson E, Hutchinson R, Ward H, 2018. Tolerance of uncertainty: A systematic review of health and healthcare-related outcomes. Patient Education and Counseling 101 (9): 1518–1537. 10.1016/j.pec.2018.03.030 29655876

[8] Hancock J, and Mattick K. 2020. Tolerance of ambiguity and psychological well-being in medical training: A systematic review. Medical Education 54 (2): 125–137. 10.1111/medu.14031 31867801 PMC7003828

[9] Begin AS, Hidrue MK, Lehrhoff S, Lennes IT, Armstrong K, Weilburg JB, 2022. Association of self-reported primary care physician tolerance for uncertainty with variations in resource use and patient experience. JAMA Network Open 5 (9) : e2229521. 10.1001/jamanetworkopen.2022.29521 36048444 PMC9437748

[10] Luther VP, and Crandall SJ. 2011. Commentary: Ambiguity and uncertainty: Neglected elements of medical education curricula? Academic Medicine 86 (7): 799–800. 10.1097/ACM.0b013e31821da915 21715991

[11] Patel P, Hancock J, Rogers M, and Pollard SR. 2022. Improving uncertainty tolerance in medical students: A scoping review. Medical Education 56 (12): 1163–1173. 10.1111/medu.14873 35797009 PMC9796811

[12] Stephens GC, and Lazarus MD. 2024. Twelve tips for developing healthcare learners’ uncertainty tolerance. Medical Teacher 46 (8): 1035–1043. 10.1080/0142159X.2024.2307500 38285073

[13] Geller G 2013. Tolerance for ambiguity: An ethics-based criterion for medical student selection. Academic Medicine 88 (5): 581–584. 10.1097/ACM.0b013e31828a4b8e 23524934

[14] Hancock J, and Mattick K. 2012. Increasing students’ tolerance of ambiguity: the need for caution. Academic Medicine 87 (7): 834. 10.1097/ACM.0b013e318257d085 22735554

[15] Ilgen JS, Watsjold BK, and Regehr G. 2022. Is uncertainty tolerance an epiphenomenon? Medical Education 56 (12): 1150–1152. 10.1111/medu.14938 36124815

[16] Haas M, and Stojan JN. 2022. Uncertainty about uncertainty tolerance: The elephants in the room. Medical Education 56 (12): 1152–1154. 10.1111/medu.14926 35980941

[17] Reis-Dennis S, Gerrity MS, and Geller G. 2021. Tolerance for uncertainty and professional development: A normative analysis. Journal of General Internal Medicine 36 (8): 2408–2413. 10.1007/s11606-020-06538-y 33532966 PMC7853704

[18] Han PKJ 2021. Uncertainty in medicine: A framework for tolerance. New York: Oxford University Press.

[19] Han PKJ 2021. Medical uncertainty: Putting flesh on the bones. Patient Education and Counseling 104 (11): 2603–2605. 10.1016/j.pec.2021.09.001 34666906 PMC8520411

[20] Durrheim K, and Foster D. 1997. Tolerance of ambiguity as a content specific construct. Personality and Individual Differences 22:741–750. 10.1016/S0191-8869(96)00207-3

[21] Herman JL, Stevens MJ, Bird A, Mendenhall M, and Oddou G. 2010. The tolerance for ambiguity scale: Towards a more refined measure for international management research. International Journal of Intercultural Relations 34 (1): 58–65. 10.1016/J.IJINTREL.2009.09.004

[22] Furnham A, and Ribchester T. 1995. Tolerance of ambiguity: A review of the concept, its measurement and applications. Current Psychology 14 (3): 179–199. 10.1007/BF02686907

[23] Grenier S, Barrette A-M, and Ladouceur R. 2005. Intolerance of uncertainty and intolerance of ambiguity: Similarities and differences. Personality and Individual Differences 39 (3): 593–600. 10.1016/j.paid.2005.02.014

[24] Birrell J, Meares K, Wilkinson A, and Freeston M. 2011. Toward a definition of intolerance of uncertainty: A review of factor analytical studies of the Intolerance of Uncertainty Scale. Clinical Psychology Review 31 (7): 1198–1208. 10.1016/j.cpr.2011.07.009 21871853

[25] Furnham A, and Marks J. 2013. Tolerance of ambiguity: A review of the recent literature. Psychology 4:717–728. 10.4236/psych.2013.49102

[26] Rosen NO, Ivanova E, and Knauper B. 2014. Differentiating intolerance of uncertainty from three related but distinct constructs. Anxiety, Stress, & Coping 27 (1): 55–73. 10.1080/10615806.2013.815743 23849047

[27] McLain DL, Kefallonitis E, and Armani K. 2015. Ambiguity tolerance in organizations: Definitional clarification and perspectives on future research. Frontiers in Psychology 6: 344. 10.3389/fpsyg.2015.00344 25972818 PMC4411993

[28] Carleton R, Collimore K, and Asmundson G. 2010. “It’s not just the judgements–It’s that I don’t know”: Intolerance of uncertainty as a predictor of social anxiety. Journal of Anxiety Disorders 24:189–195. 10.1016/j.janxdis.2009.10.007 19931391

[29] Norton RW 1975. Measurement of ambiguity tolerance. Journal of Personality Assessment. 39 (6): 607–619. 10.1207/s15327752jpa3906_11 16367289

[30] Budner S 1962. Intolerance of ambiguity as a personality variable. Journal of Personality. 30 (1): 29–50. 10.1111/j.1467-6494.1962.tb02303.x 13874381

[31] Carleton RN 2016. Fear of the unknown: One fear to rule them all? Journal of Anxiety Disorders 41:5–21. 10.1016/j.janxdis.2016.03.011 27067453

[32] Mahoney AEJ, and McEvoy PM. 2012. A transdiagnostic examination of intolerance of uncertainty across anxiety and depressive disorders. Cognitive Behaviour Therapy 41 (3): 212–222. 10.1080/16506073.2011.622130 22032195

[33] Robichaud M, Koerner N, and Dugas MJ. 2019. Cognitive behavioral treatment for generalized anxiety disorder. New York: Routledge.

[34] Geller G, Grbic D, Andolsek KM, Caulfield M, and Roskovensky L. 2021. Tolerance for ambiguity among medical students: patterns of change during medical school and their implications for professional development. Academic Medicine 96 (7): 1036–1042. 10.1097/ACM.0000000000003820 33149092

[35] Stephens GC, Rees CE, and Lazarus MD. 2021. Exploring the impact of education on preclinical medical students’ tolerance of uncertainty: A qualitative longitudinal study. Advances in Health Sciences Education 26 (1): 53–77. 10.1007/s10459-020-09971-0 32378150

[36] Beck U 1992. Risk Society : Towards a New Modernity. London; Newbury Park, Calif.: Sage Publications; 1992.

[37] Skolbekken JA 1995. The risk epidemic in medical journals. Social Science & Medicine 40 (3): 291–305. 10.1016/0277-9536(94)00262-r 7899942

[38] Canguilhem G 1989. The normal and the pathological. New York: Zone Books.

[39] Carver CS, and Scheier MF. 1982. Control theory: A useful conceptual framework for personality-social, clinical, and health psychology. Psychological Bulletin 92 (1): 111–135. 10.1037/0033-2909.92.1.111 7134324

[40] Carver CS, and Scheier M. 1998. On the self-regulation of behavior. New York: Cambridge University Press.

[41] Carver CS, and Scheier MF. 2000. On the structure of behavioral self-regulation. In Handbook of self-regulation, ed. Boekaerts M, Pintrich PR, and Zeidner M, 41–84. Academic Press.

[42] Kawall J 2009. In defence of the primacy of virtues. Journal of Ethics and Social Philosophy. 3 (2): 1–21.

[43] MacIntyre A 1979. Designing our descendants. Seven traits for the future. Hastings Center Report 9 (1): 5–7. 10.2307/3561692 429065

[44] McGrath RE, and Brown M. 2020. Using the VIA classification to advance a psychological science of virtue. Frontiers in Psychology 11 : 565953. 10.3389/fpsyg.2020.565953 33364995 PMC7751260

[45] McGrath RE 2021. Darwin meets Aristotle: Evolutionary evidence for three fundamental virtues. Journal Of Positive Psychology 16 (4): 431–445. 10.1080/17439760.2020.1752781

[46] Peterson C, and Seligman MEP. 2004. Character strengths and virtues: A handbook and classification. New York: Oxford University Press.

[47] Schwartz B, and Sharpe KE. 2006. Practical wisdom: Aristotle meets positive psychology. Journal of Happiness Studies 7 (3): 377–395. 10.1007/S10902-005-3651-Y

[48] Han PK, Klein WM, and Arora NK. 2011. Varieties of uncertainty in health care: A conceptual taxonomy. Medical Decision Making 31 (6): 828–838. 10.1177/0272989x11393976 22067431 PMC3146626

[49] Ng V, and Tay L. 2020. Lost in translation: The construct representation of character virtues. Perspectives on Psychological Science 15 (2): 309–326. 10.1177/1745691619886014 31971864

[50] Fowers BJ, Carroll JS, Leonhardt ND, and Cokelet B. 2021. The emerging science of virtue. Perspectives on Psychological Science 16 (1): 118–147. 10.1177/1745691620924473 32835627

[51] Vallor S 2016. Technology and the Virtues : A Philosophical Guide to a Future Worth Wanting. New York, NY: Oxford University Press.

[52] Crawford-Brown DJ 1997. Virtue as the basis of engineering ethics. Science and Engineering Ethics 3 (4): 481–489. 10.1007/s11948-997-0049-8

[53] Harris CE Jr. 2008. The good engineer: Giving virtue its due in engineering ethics. Science and Engineering Ethics 14 (2): 153–164. 10.1007/s11948-008-9068-3 18461475

[54] Schmidt JA 2014. Changing the paradigm for engineering ethics. Science And Engineering Ethics 20 (4): 985–1010. 10.1007/s11948-013-9491-y 24189836

[55] Frigo G, Marthaler F, Albers A, Ott S, and Hillerbrand R. 2021. Training responsible engineers. Phronesis and the role of virtues in teaching engineering ethics. Australasian Journal of Engineering Education 26 (1): 25–37. 10.1080/22054952.2021.1889086

[56] Bergen JP, and Robaey Z. 2022. Designing in times of uncertainty: what virtue ethics can bring to engineering ethics in the twenty-first century. In Values for a Post-Pandemic Future, ed. Dennis MJ, Ishmaev G, Umbrello S, and van den Hoven J, 163–183. Cham: Springer International Publishing. 10.1007/978-3-031-08424-9_9

[57] Kaldjian LC 2014. Practicing Medicine and Ethics : Integrating Wisdom, Conscience, and Goals of Care. New York: Cambridge University Press.

[58] Arthur J, Kristjánsson K, Thomas H, Kotzee H, Ignatowicz A, and Qiu T. 2015. Virtuous Medical Practice. Jubilee Centre for Character and Virtues: University of Birmingham. http://www.jubileecentre.ac.uk/1555/projects/gratitudebritain/virtuous-medical-practice

[59] Kotzee B, Ignatowicz A, and Thomas H. 2017. Virtue in medical practice: An exploratory study. HEC Forum 29 (1): 1–19. 10.1007/s10730-016-9308-x 27557996 PMC5306150

[60] Pellegrino ED, and Thomasma DC. 1993. The Virtues in Medical Practice. New York: Oxford University Press.

[61] Jameel SY 2025. A critical interpretive literature review of phronesis in medicine. Journal of Medicine and Philosophy 50 (2): 117–132. 10.1093/jmp/jhae045 39970275 PMC11925555

[62] Miller CB 2025. Challenges Facing the Appeal to Practical Wisdom in Medicine and Beyond. Journal of Medicine and Philosophy 50 (2): 93–103. 10.1093/jmp/jhae047 39776170 PMC11925554

[63] Huber A, Strecker C, Kachel T, Hoge T, and Hofer S. 2020. Character strengths profiles in medical professionals and their impact on well-being. Frontiers In Psychology 11 : 566728. 10.3389/fpsyg.2020.566728 33424679 PMC7786021

[64] Sfara E 2023. From technique to normativity: The influence of Kant on Georges Canguilhem’s philosophy of life. History and Philosophy of the Life Sciences. 45 (2): 1–33. 10.1007/s40656-023-00573-8 37022509 PMC10284956

[65] Aristotle. 2004. The Nicomachean Ethics. (Thomson JAK, Transl.). New York: Penguin Books (Original work published ca. 350 B.C.E.).

[66] Baehr JS 2011. The Inquiring Mind : On Intellectual Virtues and Virtue Epistemology. Oxford: Oxford University Press.

[67] Porter T, Baldwin CR, Warren MT, Murray ED, Cotton Bronk K, Forgeard MJC, 2022. Clarifying the content of intellectual humility: A systematic review and integrative framework. Journal Of Personality Assessment 104 (5): 573–585. 10.1080/00223891.2021.1975725 34569872

[68] Tillich P 1952. The courage to be. New Haven: Yale University Press.

[69] Han PK 2013. Conceptual, methodological, and ethical problems in communicating uncertainty in clinical evidence. Medical Care Research and Review 70 (1 Suppl): 14S–36S. 10.1177/1077558712459361 23132891 PMC4238424

[70] Han PK 2016. The need for uncertainty: A case for prognostic silence. Perspectives in Biology and Medicine 59 (4): 567–575. 10.1353/pbm.2016.0049 28690246 PMC5560765

[71] Winkler RL 1991. Ambiguity, probability, preference, and decision analysis. Journal of Risk and Uncertainty 4:285–297. 10.1007/BF00114158

[72] Han PK, Klein WM, Lehman TC, Massett H, Lee SC, and Freedman AN. 2009. Laypersons’ responses to the communication of uncertainty regarding cancer risk estimates. Medical Decision Making 29 (3): 391–403. 10.1177/0272989X08327396 19470720 PMC2730504

[73] Han PKJ, Gutheil C, Hutchinson RN, and LaChance JA. 2020. Cause or effect? The role of prognostic uncertainty in the fear of cancer recurrence. Frontiers In Psychology 11 : 626038. 10.3389/fpsyg.2020.626038 33519656 PMC7843433

[74] Han PKJ, Scharnetzki E, Scherer AM, Thorpe A, Lary C, Waterston LB, 2021. Communicating scientific uncertainty about the COVID-19 pandemic: online experimental study of an uncertainty-normalizing strategy. Journal of Medical Internet Research 23 (4) : e27832. 10.2196/27832 33769947 PMC8064708

[75] Han PKJ, Scharnetzki E, Anderson E, DiPalazzo J, Strout TD, Gutheil C, 2022. Epistemic beliefs: Relationship to future expectancies and quality of life in cancer patients. Journal of Pain and Symptom Management 63 (4): 512–521. 10.1016/j.jpainsymman.2021.12.017 34952170 PMC8930513

[76] Wright JC, Warren M, and Snow NE. 2020. Understanding Virtue : Theory and Measurement. New York, NY, United States of America: Oxford University Press.

[77] Hofer BK, Pintrich PR Personal Epistemology : The Psychology of Beliefs About Knowledge and Knowing. Mahwah, N.J.: L. Erlbaum Associates; 2002.

[78] Bendixen LD, Feucht FC. Personal Epistemology in the Classroom : Theory, Research, and Implications for Practice. Cambridge, UK; New York: Cambridge University Press; 2010.

[79] Sosu EM 2013. The development and psychometric validation of a critical thinking disposition scale. Thinking Skills and Creativity. 9:107–119. 10.1016/j.tsc.2012.09.002

[80] Sahdra BK, Shaver PR, and Brown KW. 2010. A scale to measure nonattachment: A Buddhist complement to Western research on attachment and adaptive functioning. Journal of Personality Assessment 92 (2): 116–127. 10.1080/00223890903425960 20155561

[81] Merluzzi TV, and Philip EJ. 2017. “Letting Go”: From ancient to modern perspectives on relinquishing personal control-a theoretical perspective on religion and coping with cancer. Journal of Religion and Health 56 (6): 2039–2052. 28168581 10.1007/s10943-017-0366-4

[82] Merluzzi TV, Salamanca-Balen N, Philip EJ, and Salsman JM. 2023. “Letting go” - Relinquishing control of illness outcomes to God and quality of life: Meaning/peace as a mediating mechanism in religious coping with cancer. Social Science & Medicine 317 : 115597. 10.1007/s10943-017-0366-4 36535230 PMC9962851

[83] Baehr JS 2016. Intellectual virtues and education: Essays in applied virtue epistemology. New York: Routledge, Taylor & Francis Group.

[84] Hafferty FW 1998. Beyond curriculum reform: Confronting medicine’s hidden curriculum. Academic Medicine 73 (4): 403–407. 10.1097/00001888-199804000-00013 9580717

[85] Silveira GL, Campos LKS, Schweller M, Turato ER, Wong A, and Trollope-Kumar K. 2014. Reflections: An inquiry into medical students’ professional identity formation. Medical Education 48:489–501. 10.1111/medu.12382 24712934

[86] Sawatsky AP, Matchett CL, Hafferty FW, Cristancho S, Bynum WE, Ilgen JS, and Varpio L. 2024. Identity work: A qualitative study of residents’ experiences navigating identity struggles. Perspectives on Medical Education 13 (1): 540–552. 10.5334/pme.1549 39554488 PMC11568810

[87] Fraser SW, and Greenhalgh T. 2001. Coping with complexity: Educating for capability. BMJ 323 (7316): 799–803. 10.1136/bmj.323.7316.799 11588088 PMC1121342

